# Effects of Different Pilot-Scale Milling Methods on Bioactive Components and End-Use Properties of Whole Wheat Flour

**DOI:** 10.3390/foods10112857

**Published:** 2021-11-18

**Authors:** Wenfei Tian, Jingyang Tong, Xiaoyue Zhu, Philipp Fritschi Martin, Yonghui Li, Zhonghu He, Yan Zhang

**Affiliations:** 1 National Wheat Improvement Centre, Institute of Crop Sciences, Chinese Academy of Agricultural Sciences, Beijing 100081, China; tianwenfei@caas.cn (W.T.); 82101192196@caas.cn (J.T.); hezhonghu02@caas.cn (Z.H.); 2 International Maize and Wheat Improvement Center (CIMMYT) China Office, Chinese Academy of Agricultural Sciences, Beijing 100081, China; 3 Grain Technology Center, Buhler (Wuxi) Commercial Co., LTD., Wuxi 214142, Jiangsu, China; arena.zhu@buhlergroup.com (X.Z.); philipp.fritschi@buhlergroup.com (P.F.M.); 4 Department of Grain Science and Industry, Kansas State University, Manhattan, KS 66506, USA; yonghui@ksu.edu

**Keywords:** milling methods, dietary fiber, phenolic acid, steamed bread, leavened pancake

## Abstract

The health benefits from consumption of whole wheat products are widely recognized. This study investigated the effects of different pilot-scale milling methods on physicochemical properties, bioactive components, Chinese steamed bread (CSB), and Chinese leavened pancakes (CLP) qualities of whole wheat flour (WWF). The results indicated that WWF-1 from the reconstitution of brans processed by a hammer mill had the best CSB and CLP quality overall. WWF from entire grain grinding by a jet mill (65 Hz) contained the highest concentration of bioactive components including dietary fibers (DF) and phenolic acids. A finer particle size did not necessarily result in a higher content of phenolic antioxidants in WWF. DF contents and damaged starch were negatively correlated with CSB and CLP quality. Compromised reduced quality observed in CLP made from WWF indicated its potentially higher acceptance as a whole-grain product.

## 1. Introduction

Bread wheat (*Triticum aestivum* L.) is among the most widely planted cereal crops [1]. Consumption of whole-grain products has been associated with reduced risk of chronic diseases, such as cardiovascular disease, type 2 diabetes, obesity, and some types of cancer [2,3]. The health benefits of whole-grain products can be partially attributed to their contents of dietary fiber (DF) and phytochemicals, such as phenolic acids and phytosterols [4,5]. Phenolic acids have promising antioxidative, anti-inflammatory, and antimicrobial activities [6,7].The content of phenolic compounds is becoming another parameter evaluating the quality of the whole wheat flour [8,9]. Although whole-wheat flour (WWF) can provide high amounts of DF and other bioactive components, products made from it remain under-utilized globally. This can be partially attributed to poorer sensory properties, a shorter shelf life, and a higher risk of rancidity [10,11]. The proper use of enzymes, emulsifiers, and hydrocolloids can improve the sensory properties of the whole wheat products [12]. However, the common whole-wheat improvers, such as diacetyl tartaric acid ester of mono- and diglycerides (DATEM) and sodium stearoyl lactylate (SSL), cannot pass the clean label test which may limit their application in food industry in the future. Different milling techniques can also influence the technical and end-use properties of WWF, possibly by modifying its particle sizes and chemical composition [13]. A good amount of study have indicated the effects of bran particle sizes on the quality of WWF [14,15,16,17,18]. Obviously, different bran particle sizes come from different milling methods including different types of the mills and rotor speeds. Therefore, it is necessary to develop proper milling methods to produce WWF products with enhanced sensory properties. Currently, most commercial WWF is produced using roller mills. The roller mill separates the endosperm fraction from the coarse bran and germ fraction. Coarse brans are usually micronized and then re-mixed with the endosperm for the production of WWF. Hammer mills (HM) and jet mills (JM) can be used for bran micronization [19]. WWF can also be prepared by direct pulverization of entire wheat grains using jet mills without separation and reconstitution of the components [19,20]. However, this method may result in a higher content of damaged starch. It is important to investigate the effects of this entire grain grinding method on the quality of the resulting WWF.

Chinese steamed bread (CSB) is a traditional fermented and steamed food in China with distinctive cultural features across the country [21]. It is a staple food in northern China with increasing popularity beyond [22]. There are many significant differences between CSB and western-style baked bread owing to the different production procedures. For instance, the lower processing temperature (100 °C) for CSB might permit higher preservation of diverse endogenous nutrients and reduce the production of toxic Maillard reaction products, such as acrylamide and furans [23]. Further, the general ingredients of CSB only include flour, water, and yeast. The absence of added sugar, oil, and salt renders CSB additional health benefits. Chinese leavened pancake (CLP) is another popular wheat product consumed as a staple food in some parts of China. However, previous literature on the quality of CLP made from WWF is not available. Compared to western-style whole wheat bread, factors affecting quality of whole wheat CSB and CLP are under-investigated. It was reported that finer particle sizes increased the specific volume and improved the crumb texture of CSB [15]. Bran dietary fibers and phenolics also influenced the CSB quality. As for the effect of milling process, to our knowledge, there was only one study whole results suggested that CSB from bran reconstitution had larger loaf volume, but the entire grain grinding improved the color of the CSB [19]. More studies are needed to gain further understanding on the quality of CSB and CLP. Furthermore, the effects of different milling methods on the bioactive components, such as dietary fiber and phenolic acids of WWF, are also not thoroughly investigated. Due to their potential health benefits [24,25], the content of total phenolics is becoming another factor determining wheat market preference [26]. In addition, previous studies were conducted using lab-scale mills. Information at pilot-scale level is very limited. Therefore, the objective of this study was to systematically investigate the effects of different pilot-scale milling methods on bioactive components (including dietary fiber and phenolic acids), physicochemical properties of WWF, as well as its effect on CSB and CLP quality. The outcome of the study provides useful information for the quality improvement of WWF products and contributes to the facilitation of high-DF food consumption in enhancing health benefits for consumers.

## 2. Materials and Methods

### 2.1. Wheat Grains and Chemicals

A sound grain mixture (1:1) of wheat cultivars Zhongmai 578 (1000 kg) and Zhongmai 175 (1000 kg) was used for preparation of WWF. Zhongmai 578 (farinograph stability time = 23.6 min, wet gluten = 34.2%) is an important cultivar in the Yellow-Huai river winter wheat region of China. Zhongmai 175 (farinograph stability time = 2.0 min, wet gluten = 27.4%) is the most widely grown cultivar in the northern China plain winter wheat region. General chemicals, HPLC grade water, HPLC grade acetonitrile, and analytical standards (including vanillic acid, syringic acid, *para*-coumaric acid, *trans*-ferulic acid, and sinapic acid) were purchased from Aladdin Biochemical Technology Co., Ltd. (Shanghai, China). The instant dry yeast was purchased from Lesaffre (Marcq-en-Barœul, France). The colza oil was purchased from Luhua Group Co., Ltd. (Yantai, China).

### 2.2. Pilot-Scale Milling Methods for Producing Whole Wheat Flour

After cleaning and tempering to 16.0% moisture for 28 h, the mixed grain sample of 2000 kg was milled on a Buhler roller mill located at Buhler Commercial Co. Ltd. (Wuxi, China). The mill was equipped with four break sections (B1, B2, B3, B4) and four reduction sections (R1, R2, R3, R4). During the process, a portion of crushed wheat from B1 stream (200 kg) were taken out, divided equally into two parts and then directly pulverized by jet mill (AHFL, Buhler Commercial Co. Ltd., Wuxi, China) with the rotor speeds of 45 Hz (JM-45 Hz) and 65 Hz (JM-65 Hz). The WWF from JM-45 Hz and JM-65 Hz were designated as WWF-4 and WWF-5, respectively. The other grains of 1800 kg went through the whole roller mill process and the yield of the refined flour was 72%. The bran and germ fractions from the roller mill were collected, equally divided into three parts, and then micronized using a hammer mill (AHZCφ1 mm, Buhler Commercial Co. Ltd., Wuxi, China) and a jet mill (AHFL, Buhler Commercial Co. Ltd., Wuxi, China) with the rotor speeds of 45 Hz (JM-45 Hz) and 65 Hz (JM-65 Hz), respectively. The micronized wheat bran was remixed with the refined flour at a ratio of 28:72 (bran of 28 kg, refined flour of 72 kg) to obtain WWF samples designated as WWF-1 (bran micronized by HM), WWF-2 (bran micronized by JM45-Hz), and WWF-3 (bran micronized by JM-65 Hz). For each WWF, two batches were prepared. (Supplementary Document, Table S1).

### 2.3. Analysis of Particle Size Distribution

Particle size distribution was determined using an on-line particle analyzer (MYTA, Buhler, Switzerland) equipped with a dry sample delivery and measurement system that combined laser diffraction and image processing. The system was equipped with a series of sieves with particle sizes ranging from 10 to 5000 µm. During the measurement, the percentage of flours passing through the certain sieve size was determined. Mean particle sizes (MPS) were determined as:

MPS = ∑ sieve average particle size (μm) × percentage of particles that pass through the sieve (Through X%). Original data are provided in the Supplementary Documents.

### 2.4. Analysis of Chemical Composition

Starch damage was determined using a rapid method with a SDmatic analyzer (Chopin Technologies, Paris, France) according to AACC International Approved Method 76-33.01 [27]. The method measured the amount of iodine absorbed by the tested sample in a liquid suspension. Higher content of damaged starch led to more fixed iodine. Protein content was recorded with a Foss-Tecator 1241 (Foss, Högänas, Sweden) NIR analyzer. Contents of soluble dietary fiber (SDF) and insoluble dietary fiber (IDF) were analyzed according to the Chinese national standard method GB 5009.88-2014 [28] which was very similar to the AACC approved method 32-07.01 [29]. Briefly, the flour was treated by sequentialenzymatic hydrolysis using heat-stable alpha-amylase, protease, and amyloglucosidase. IDF was filtered, and the residue was washed with warm distilled water and weighed. For SDF, the filtrate and water washing from the above step were precipitated with 95% ethanol, and the residue was then filtered, dried, and weighed. Determination of fatty acids contents followed Chinese national standard GB/T 5510-2011 [30]. Briefly, the free fatty acids were extracted with benzene and titrated with KOH solution.

### 2.5. Phenolic Acid Composition of Whole Wheat Flour

The phenolic acids in WWF were extracted according to a method reported previously [31] with some modifications. Briefly, WWF (1 g) was hydrolyzed with 2 M of NaOH (10 mL) for 4 h under nitrogen protection in darkness. The mixture was then acidified to pH 1 using 6 M of HCl and extracted three times with ethyl acetate. The combined extract was evaporated to dryness and re-dissolved in HPLC grade methanol (2 mL). The final extract was filtered through a 0.22-µm filter and analyzed within 12 h.

A UPLC-PDA system from Waters Corporation (Milford, MA, USA) equipped with an ACQUITY UPLC BEH C18 (2.1 mm × 50 mm) column was used to analyze phenolic acids following a previously reported gradient protocol [32] with some modifications. Briefly, mobile phase A comprised HPLC-grade water containing 0.1% trifluoroacetic acid (TFA), and mobile phase B was HPLC-grade acetonitrile with 0.1% TFA. The PDA detector was set to record the spectra information from 210 nm to 400 nm and also the absorption at 280 nm. The flow rate of the mobile phase was kept at 0.4 mL/min and the percentage of mobile phase B was kept at 6% from 0–1.0 min, which then increased linearly to 14% from 1.0 min to 3.0 min and increased linearly to 18% from 3.0 min to 5.5 min. The column was re-equilibrated for 2 min between each injection. Phenolic acids were identified by comparison with the retention time of analytical standards and quantified using external calibration curves using the absorbance at 280 nm. *Cis*-ferulic acid was identified according to a previous study [31] and tentatively quantified according to the calibration of *trans*-ferulic acid since the analytical standard of *cis*-ferulic acid was not available.

### 2.6. Analysis of Dough Properties

Dough rheological properties were determined by Farinograph and Extensograph (Brabender, Duisburg, Germany), following the AACC International Approved Methods 54-21.02 [33] and 54-10.01 [34], respectively. Water absorption (%), dough development time (min), and stability (min) were measured by Farinograph. Dough energy (cm^2^), extensibility (mm), and maximum resistance (BU) were determined by Extensograph.

### 2.7. Preparation and Sensory Evaluation of Chinese Steamed Bread

CSB was prepared and evaluated according to a previous study [35], with minor modifications. In brief, WWF (100 g) was mixed with yeast (2 g) and water for two minutes in a National mixer (National MFG. Co, Lincoln, NL, USA). The mixer speed was set at 90 rpm and the water addition was set at 80% of optimal water absorption (%) pre-determined by Farinograph analysis in preliminary tests. For example, if the optimal water absorption was determined as 70% by the Farinograph, we would use 56% as the optimal water level for CSB making. Fairnograph is the technique developed for western-style bread making use of refined flours. The dough was then sheeted by passing through a pair of rollers for 10 times. After each pass, the sheeted dough was folded along the side and rotated through 90°. The dough piece was then rounded five times with a suitably sized bowl. The rounded dough was proofed for 20 min in a fermentation cabinet (35 °C, 85% RH) and steamed for 25 min in a steamer initially containing cold water.

The CSB score was a weighted value based on specific volume (20), skin color (10), smoothness (10), shape (10), structure (15), and stress relaxation (35), according to the method proposed previously. Specific volume, skin color, and stress relaxation were based on objective measurements with sufficient details provided in the Supplementary Documents. Specific volume indicated the loaf volume and weight ratio of CSB. Loaf volume was determined by the rapeseed displacement method, according to AACC Approved Method 10-05.01. Skin color was measured with a Minolta CR 310 chromameter (Minolta Camera, Osaka, Japan). Skin color score was calculated according to the method reported previously [36]. Stress relaxation was measured with a TA-XT2i texture analyzer (Stable Micro Systems, Surrey, Godalming, UK). Briefly, after 15 min of steaming, two slices (3 cm thick) were cut from the center of the CSB. Each slice was placed on a flat metal plate and compressed twice to 50% of its original thickness at a speed of 1.0 mm/s with a cylindrical P35 probe. The calculation and scoring of stress relaxation was conducted according to a previous report [36]. The smoothness, shape, and structure of steamed bread were scored subjectively by five trained panelists. A high score for smoothness was given for very smooth skin; freedom of wrinkles, dimples, blisters, or gelatinized spots; and a round shape and fine crumb structure combined with uniform porosity contributed to high scores for shape and structure, respectively.

### 2.8. Preparation and Sensory Evaluation of Chinese Leavened Pancakes

To prepare CLP, WWF (100 g) was mixed with 120 mL of water (35 °C), 5 g sugar, 1 g of salt, 2 g of yeast, and 16 g of colza oil in a National mixer for 1.5 min (National MFG. Co, Lincoln, NE, USA). The mixer speed was set at 90 rpm. The dough was proofed for 1 h in a fermentation cabinet (35 °C, 85% RH). The fermented dough was gently kneaded by hand to form a rounded piece with a smooth upper surface. The rounded piece was flattened using a rolling pin to produce a 1-cm-thick dough sheet and then rested for 15 min at room temperature. The dough sheet was finally cooked for 8 min in an electric baking pan (MC-JS 3406, Midea Life Appliance Manufacturing Co., Ltd., Foshan, China). To our knowledge, there is not an international method on sensory evaluation of CLP. In this study, the weighted CLP score included appearance (20), inner structure (20), stress relaxation (35), stickiness (15), and taste and flavor (10). Stress relaxation was measured with a TA-XT2i texture analyzer (Stable Micro Systems, Surrey, Godalming, UK) according to the same method as CSB. Appearance, inner structure, stickiness, and taste and flavor of CLP were scored subjectively by five trained panelists. A high score for appearance was given to pale yellow skin, round shape, and freedom of large black spots. A fine crumb with uniform porosity and non-sticking contributed to high scores for inner structure and stickiness, respectively. Freedom from bad smell and a rich wheat flavor related to high scores for taste and flavor.

### 2.9. Short-Term Storage Quality of Chinese Steamed Bread

Texture profiles of CSB from WWF were determined after storage for 6, 12, 24, 48, and 72 h at room temperature using a TA-XT2i texture analyzer. Briefly, two slices (3 cm thick) were cut from the center of the CSB. Each slice was placed on a flat metal plate and compressed twice to 50% of its original thickness at a speed of 1.0 mm/s with a cylindrical P35 probe. Firmness and resilience were recorded.

### 2.10. Statistical Analyses

For chemical analyses, such as determination of phenolic acid composition, the results were reported as mean values from three replicates. For dough property parameters, the results were reported as mean values from two replicates. Evaluation of CSB and CLP were conducted by five trained panelists and reported as mean scores from the five panelists. The results were reported as mean ± standard deviation (SD). Results were subjected to one-way analysis of variance (AVONA) and Turkey’s test using SAS software, version 9.3 (Cary, NC, USA). *p* < 0.05 was considered as significantly different.

## 3. Results and Discussions

The quality of wheat flour is evaluated according to their chemical composition including protein content, damaged starch, and bioactive components (such as phenolic acids and dietary fibers). Dough rheological properties can be evaluated with a farinograph and an extensograph. Particle size is an important factor affecting the end-use properties of whole wheat flour.

### 3.1. Particle Sizes and Chemical Compositions of Whole Wheat Flours

Particle size distribution is an important parameter affecting end-use quality of whole wheat flour [17]. Particle size distributions of WWFs from different milling process are plotted in Figure S1 in the Supplementary Documents. Mean particle sizes (MPS) for WWF-5 were 236 µm, 146 µm, 124 µm, 191 µm and 146 µm, respectively. The jet milling was more effective than the hammer milling in reducing bran particle size. Chemical compositions of the WWFs are presented in Table 1. WWF-3 and WWF-5 from the JM-65 Hz contained higher contents of total dietary fibers (TDF) and damaged starch than the other WWFs. The higher content of damaged starch can be attributed to the higher rotor speed. Interestingly, there was a strong positive correlation between the content of damaged starch content and total DF (R^2^ = 0.7974). WWF-5 had a significantly lower (*p* < 0.05) protein content than other WWFs. WWF-3 contained the highest content of fatty acids, a factor that can lead to rancidity of food products during storage [37].

### 3.2. Phenolic Acid Compositions of Whole Wheat Flour

**Figure 1:**
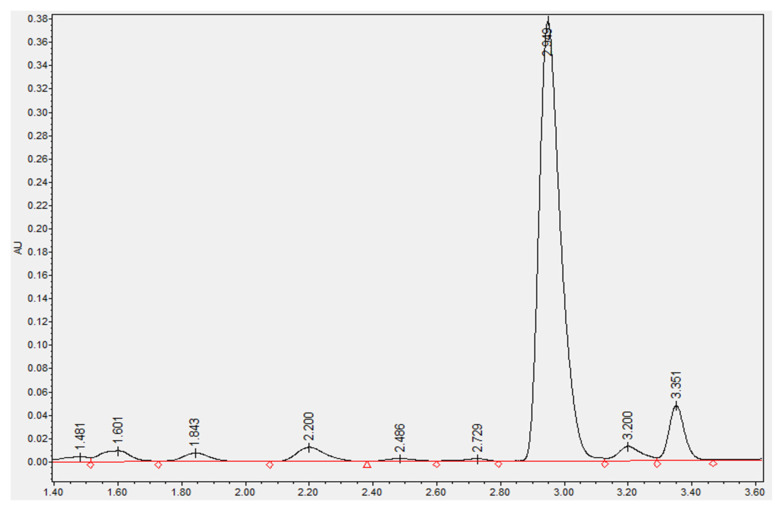
A typical UPLC spectra of the tested sample at 280 nm.

Phenolic acids are a major type of bioactive phytochemical in whole wheat [38,39]. Antioxidant and anti-inflammatory activities of phenolic acid are widely recognized [40,41]. Vanillic acid, syringic acid, *para*-coumaric acid, *trans*-ferulic acid, sinapic acid, and *cis*-ferulic acid were isolated and quantified. A typical UPLC spectra was presented in Figure 1. The retention time for vanillic acid, syringic acid, *para*-coumaric acid, *trans*-ferulic acid, sinapic acid, and *cis*-ferulic acid were 1.60, 1.84, 2.20, 2.95, 3.20, and 3.55, respectively. *Trans*-ferulic acid was the predominant phenolic acid (Table 2).

WWF-1 and WWF-2 contained similar concentrations of *trans*-ferulic acids (720.16 and 742.15 µg/g, respectively), but their particle sizes were significantly different (*p* < 0.05). Although WWF-3 and WWF-5 were both processed by the JM-65 Hz mill, they had comparable particle size distributions and dietary fiber contents; WWF-3 contained the lowest (534.90 µg/g) and WWF-5 contained the highest (1002.11 µg/g) concentrations of *trans*-ferulic acid. The high rotor speed of the JM-65 Hz on wheat bran in the bran reconstitution method (WWF-3) led to significant loss of *trans*-ferulic acid during processing. This suggests that, in the entire grain grinding method using the JM-65 Hz mill (WWF-5), the starchy endosperm physically prevents the loss of ferulic acid during reduction in particle size. As a result, WWF-5 contained the highest concentration of *trans*-ferulic acid. Similarly, WWF-5 also contained a higher content of vanillic acid, syringic acid, and *para*-coumaric acid than WWF-4. This explanation is partially supported by the fact that WWF-4 prepared by entire grain grinding using the JM 45 Hz mill had a significantly lower (*p* < 0.05) concentration of *trans*-ferulic acid than WWF-5. This was probably due to smaller particle size in WWF-5 that enhanced the extractability of phenolic acids as generally reductions in particle size enhance the extractability of phenolic acids.

Previous studies reported that reductions in bran particle size led to enhanced antioxidant capacity of wheat samples [42,43]. However, those results were obtained by in vitro methods, such as total phenolic content (TPC) and 2,2’-azino-bis(3-ethylbenzothiazoline-6-sulfonic acid) (ABTS) assays. The non-specificity and limitations of those methods, which could lead to incorrect data interpretations, were recognized in recent years [44,45]. Our analysis using a UPLC instrument on phenolic acids, i.e., the major antioxidants in WWF, indicated that a reduction in particle size does not necessarily lead to enhanced phenolic acid concentration. Different milling strategies, types of mills, and rotor speeds can produce WWFs with similar particles size but significantly different phenolic acid compositions. In summary, the milling method had a great impact on phenolic acid compositions of WWF and improved its availability with grain grinding using a JM-65 Hz procedure. To our knowledge, this is the first study reporting the high variation of total dietary fibers (1.6-fold) and ferulic acid content (1.9-fold) of WWF from different milling methods.

### 3.3. Rheological Properties of Doughs Made from Whole Wheat Flour

The effects of different milling methods on dough properties of WWF are shown in Table 3. WWF-5 had the highest water absorption (73.0%) and WWF-4 had the lowest (64.8%). This could be explained by the high DF and damaged starch contents in WWF-5, leading to higher water affinity and hydration properties [46,47,48]. Dough from WWF-4 exhibited significantly shorter (*p* < 0.05) development time than other WWF doughs. Generally, moderate development time is considered a favorable dough property. Stability time is an essential factor in evaluating dough properties. Dough from WWF-1 had the longest stability time. This can be attributed to its lowest contents of DF and damaged starch. Dough from WWF-5 had the shortest stability time due to the high content of damaged starch and DF that can lead to the formation of a weakened gluten network [49]. WWF-4 and WWF-5 from entire grain milling involved significantly less energy than the recombined WWF–3. Extensibility is another important parameter for the preparation of western-style bread. Higher extensibility is considered as a favorable dough property. Generally, entire grain milling also had negative effects on extensibility of WWF. To conclude, different milling methods had significant effects on dough rheological properties of WWF. WWF from bran reconstitution, especially WWF-1 from HM, exhibited better dough properties than WWF from entire grain grinding.

### 3.4. Sensory Properties of Chinese Steamed Bread and Chinese Leavened Pancakes

**Figure 2:**
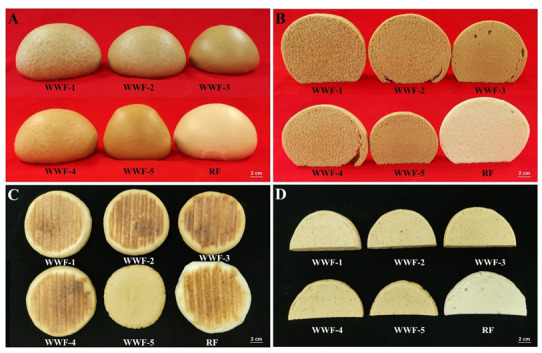
Appearance of CSB (**A**), internal structures of CSB (**B**), appearance of CLP (**C**) and internal structures of CLP (**D**) produced from different whole wheat flours. CSB: Chinese steamed bread; CLP: Chinese leavened pancakes. WWF-1: total reconstitution of brans grinded by hammer mill; WWF-2: total reconstitution of brans grinded by jet mill at 45 Hz. WWF-3: total reconstitution of brans grinded by jet mill at 65 Hz; WWF-4: entire grains grinded by jet mill at 45 Hz; WWF-5: entire grains grinded by jet mill at 65 Hz.

The CSB quality evaluations from the different WWFs presented in Table 4 and illustrated in Figure 2A,B showed that the CSB from WWF-4 had the highest total score (59), although that score was not significantly higher from the CSB score from WWF-1 (57) and WWF-2 (58). WWF-4 exhibited inferior dough properties compared to WWF-1 and WWF-2 in terms of stability, energy, and extensibility, suggesting that inferior dough properties may not affect the quality of final products. WWF-3 showed a significantly higher score than WWF-5, probably attributable to the higher damaged starch and DF contents of WWF-5 interfering with the formation of the gluten network [14,50]. Compared to CSB from WWF-1 and WWF-4, CSB from WWF-2, WWF-3, and WWF-5 received higher scores in smoothness but lower scores in specific volume. This could be due to the relatively smaller particle sizes of WWF-2, WWF-3, and WWF-5 [19]. All CSBs from WWF obtained a 0 score color as the white color is currently an essential factor that influences acceptance by consumers [21].

The scores were reported as mean values from five panelists. Within each column, values followed by different letters are significantly different (*p* < 0.05).

Sensory qualities of CLP prepared from the different flours are summarized in Table 5, and Figure 2C,D illustrates the appearance of CLP and its internal structures, respectively. CLP from WWF-1 received the highest total score (70) followed by CLP from WWF-4 (66) and WWF-2 (63). All CLP showed flat and tidy surfaces, except for that from WWF-5, which showed broken edges and very low specific volume. This was likely caused by higher DF and damaged contents [14,18].

CSB made from refined flour received a score of 93 which was 34 points higher than CSB from WWF-4 with a score of 59. In contrast, CLP from refined flour received a score of 87 which was only 17 points higher than CLP from WWF-1 with a score of 70. This result indicated a potentially higher consumer acceptance for whole-grain CLP products. Whole-grain CLP can be a promising contributor for increased whole-grain consumption in the future due to its favorable sensory properties.

### 3.5. Correlation of Sensory Quality and Chemical Composition

The general effects of damaged starch and DFs on sensory properties of whole-grain products have been extensively documented. However, to our knowledge, correlations between sensory properties and damaged starch and DF have not been widely investigated, possibly due to the lack of a comprehensive scoring system for evaluation of sensory properties. In this study, total scores for sensory evaluation as well as DF and damaged starch contents were obtained, thus paving the way for correlation analysis between certain components and end-use quality (Supplementary Document, Figure S2). Negative correlations were found between (a) the CSB score and damaged starch (R^2^ = 0.7553); (b) the CSB score and total dietary fiber (R^2^ = 0.8931); (c) the CLP score and damaged starch (R^2^ = 0.9623); and (d) the CLP score and total dietary fiber (R^2^ = 0.8375). These correlations clearly demonstrate that end-use quality of WWFs can be enhanced by reduced damaged starch content.

### 3.6. Storage Quality of CSB

Changes on texture profiles, including hardness and resilience, were measured at 6, 12, 24, 48, and 72 h storage at room temperature to assess the storage properties of CSB (Supplementary Document, Figure S3). A lower value of hardness is considered as a favorable property. At 0 h, CSB from WWF-1 had the lowest hardness (4240.5 g) while CSB from WWF-5 had the higher hardness (11,040.8 g). After 72 h of storage, the hardness of CSB from WWF-1 and WWF-5 increased to 10,519.7 g and 17,068.0 g, respectively. Generally, as shown in Figure S3A, the final hardness (72 h) of all samples showed a consistent relationship with initial hardness (0 h) as CSB from WWF-3 and WWF-5 had both high initial and final hardness, whereas that from WWF-1 had both low initial and final hardness. Compared to WWF-1 (236 µm) and WWF-4 (191 µm), WWFs with finer particle sizes, i.e., WWF-3 (124 µm) and WWF-5 (146 µm), led to the CSB products with higher hardness values. The increase in hardness during storage is probably due to recrystallization of amorphously melted starch [49]. The lower hardness of WWF-1 might result from the coarse bran particles slowing down the formation of double helix starch and retrogradation [51].

Changes in resilience are presented in Figure S3B. Resilience reflects how well a CSB slice can restore to the original height after application of pressure. A higher resilience value is generally considered preferable. CSB from WWF-1 had the highest initial resilience of 0.44, followed by CSB from WWF-4 (0.41). WWF-3 and WWF-5 had lower initial resilience, at 0.35 and 0.34, respectively. After 72 h, WWF-3 and WWF-5 maintained relatively higher resilience, at 0.24 and 0.23, respectively, while the resilience of WWF-1 and WWF-4 decreased to 0.19 and 0.19, respectively. This observation was possibly due to the fact that the degree of change in resilience is positively related to the original CSB volume [52]. Therefore, the resilience of CSB from WWF-1, WWF-2, and WWF-4 with larger original volumes decreased more dramatically than that from WWF-3 and WWF-5, both of which contained slightly higher contents of soluble dietary fiber (SDF) than other WWFs (Table 1). Rezaei et al. [53] reported that a moderately enhanced content of SDF improves the elasticity of the gluten–starch matrix and reduces starch retrogradation. The resilience of CSB from WWF-1 was comparable to that of CSB from WWF-3 and WWF-5, especially during storage of 0–48 h. By combing the data of hardness and resilience, it can be concluded that CSB from WWF-1 processed by hammer mill had the best overall storage quality over 0 to 72 h.

## 4. Conclusions

This study used five different pilot-scale milling methods to prepare whole-wheat flour.

The different milling methods had significant effects on physiochemical properties, bioactive components, and end-use properties of the WWFs. Particle size distribution was important for the quality of CSB and CLP. The content of damaged starch and dietary fiber was negatively correlated with an evaluation score of CSB and CLP. WWF-1 constituted from total bran reconstitution processed by a hammer mill exhibited best end-use properties, especially for Chinese leavened pancakes. WWF-5 from the entire grain ground by JM-65 Hz had the highest content of bioactive dietary fiber and phenolic acids, rendering its superiority in nutraceutical values. CSB from WWF-1 by total bran reconstitution using the hammer mill had the best short-term storage property. In general, WWF with better end-use properties can be obtained by the milling processes, resulting in appropriate particle size distributions and a low content of damaged starch. Although DF and phenolic acids had negative effects on sensory property of WWF, their health-promoting effects must be recognized. Different milling methods can result in WWF with significantly different contents of phenolic acids and dietary fibers. Further studies will be worthwhile for developing novel techniques that produce whole-wheat products with both high consumer acceptance and superior nutraceutical value.

## Figures and Tables

**Table 1:** Chemical composition of WWFs obtained from different milling methods.

Sample	SDF (g/100 g)	IDF (g/100 g)	TDF (g/100 g)	Fatty Acids (mg/100 g)	Damaged Starch (%)	Protein (%)
WWF-1	7.35 ± 0.16c	1.50 ± 0.24b	8.85 ± 0.07c	45.3 ± 2.26c	4.12 ± 0.06e	14.6 ± 0.06bc
WWF-2	7.27 ± 0.02c	1.73 ± 0.02ab	9.00 ± 0.05c	53.9 ± 2.61b	5.06 ± 0.01c	15.0 ± 0.04a
WWF-3	8.64 ± 0.30b	2.12 ± 0.12a	10.76 ± 0.17b	64.4 ± 2.85a	5.71 ± 0.07b	14.8 ± 0.14ab
WWF-4	6.18 ± 0.28d	1.28 ± 0.14b	7.46 ± 0.27d	42.1 ± 2.10c	4.56 ± 0.08d	14.1 ± 0.11d
WWF-5	10.24 ± 0.40a	2.03 ± 0.05a	12.27 ± 0.45a	53.0 ± 1.98b	6.35 ± 0.01a	13.5 ± 0.07e

The results were reported as mean values from three replicates. Within each column, values followed by different letters are significantly different (*p* < 0.05). WWF: whole-wheat flour; SDF: soluble dietary fiber; IDF: insoluble dietary fiber; TDF: total dietary fiber. WWF-1: total reconstitution of brans grinded by hammer mill; WWF-2: total reconstitution of brans grinded by jet mill at 45 Hz. WWF-3: total reconstitution of brans grinded by jet mill at 65 Hz; WWF-4: entire grains grinded by jet mill at 45 Hz; WWF-5: entire grains grinded by jet mill at 65 Hz.

**Table 2:** Phenolic acid composition of WWFs obtained by different milling methods.

Sample	Vanillic Acid (µg/g)	Syringic Acid (µg/g)	*para*-Coumaric Acid (µg/g)	*trans*-Ferulic Acid (µg/g)	Sinapic Acid (µg/g)	*cis*-Ferulic Acid (µg/g)
WWF-1	29.57 ± 0.80b	17.20 ± 0.68b	18.50 ± 0.81ab	720.16 ± 4.58bc	77.47 ± 1.22abc	75.08 ± 0.63b
WWF-2	31.22 ± 0.83ab	16.76 ± 0.87bc	17.82 ± 0.89ab	742.15 ± 14.62b	80.97 ± 2.10a	73.48 ± 1.40b
WWF-3	30.18 ± 1.54b	14.47 ± 0.53c	13.30 ± 0.65c	534.90 ± 6.83d	78.49 ± 2.18ab	50.81 ± 1.41c
WWF-4	27.72 ± 1.43b	14.75 ± 0.99bc	15.81 ± 0.57bc	647.33 ± 23.64c	70.65 ± 2.94c	65.77 ± 1.81b
WWF-5	34.35 ± 3.97a	19.64 ± 2.46a	19.87 ± 4.37a	1002.11 ± 101.04a	73.57 ± 6.81bc	111.25 ± 16.03a

The results were reported as mean values from three replicates. Within each column, values followed by different letters are significantly different (*p* < 0.05). WWF: whole-wheat flour. WWF-1: total reconstitution of brans grinded by hammer mill; WWF-2: total reconstitution of brans grinded by jet mill at 45 Hz. WWF-3: total reconstitution of brans grinded by jet mill at 65 Hz; WWF-4: entire grains grinded by jet mill at 45 Hz; WWF-5: entire grains grinded by jet mill at 65 Hz.

**Table 3:** Dough rheological properties of WWF obtained by different milling methods.

Sample	Water Absorption (%)	Development time (min)	Stability (min)	Energy (cm^2^)	Extensibility (mm)	Maximum Resistance (BU)
WWF-1	68.4 ± 0.1c	4.7 ± 0.0b	6.0 ± 0.1a	47.0 ± 0.3b	132.9 ± 0.5b	229.8 ± 1.8b
WWF-2	69.8 ± 0.1b	4.9 ± 0.0ab	4.7 ± 0.0b	52.8 ± 0.2a	140.0 ± 0.8a	249.0 ± 3.9a
WWF-3	68.0 ± 0.1c	5.1 ± 0.1a	4.8 ± 0.1b	49.0 ± 0.3b	142.1 ± 0.9a	227.6 ± 2.3b
WWF-4	64.8 ± 0.3d	3.8 ± 0.1c	3.8 ± 0.1c	39.5 ± 0.3c	133.6 ± 0.5b	193.0 ± 1.9c
WWF-5	73.0 ± 0.3a	4.8 ± 0.1b	2.1 ± 0.0d	38.3 ± 0.3c	105.9 ± 0.4c	243.8 ± 3.1a

The results were reported as mean values from two replicates. Within each column, values followed by different letters are significantly different (*p* < 0.05). WWF: whole-wheat flour. WWF-1: total reconstitution of brans grinded by hammer mill; WWF-2: total reconstitution of brans grinded by jet mill at 45 Hz. WWF-3: total reconstitution of brans grinded by jet mill at 65 Hz; WWF-4: entire grains grinded by jet mill at 45 Hz; WWF-5: entire grains grinded by jet mill at 65 Hz.

**Table 4:** Sensory evaluation for Chinese steamed bread produced from WWF obtained by different milling methods.

Sample	Specific Volume (20)	Stress Relaxation (35)	Skin Color (10)	Smoothness (10)	Shape (10)	Structure (15)	Total Score (100)
WWF-1	14a	21a	0a	5d	8a	9b	57a
WWF-2	14a	19b	0a	7b	7b	11a	57a
WWF-3	9b	19b	0a	8a	7b	9b	52b
WWF-4	14a	21a	0a	6c	7b	11a	59a
WWF-5	9b	13c	0a	7b	5c	8c	42c
RF	20	33	9	9	8	14	93

The scores were reported as mean values from five panelists. Within each column, values followed by different letters are significantly different (*p* < 0.05). WWF: whole-wheat flour, RF: refined flour. WWF-1: total reconstitution of brans grinded by hammer mill; WWF-2: total reconstitution of brans grinded by jet mill at 45 Hz. WWF-3: total reconstitution of brans grinded by jet mill at 65 Hz; WWF-4: entire grains grinded by jet mill at 45 Hz; WWF-5: entire grains grinded by jet mill at 65 Hz.

**Table 5:** Sensory evaluation of Chinese leavened pancakes produced from WWF obtained by different milling methods.

Sample	Appearance (20)	Stress Relaxation (35)	Structure (20)	Stickiness (15)	Taste and Flavor (10)	Total Score (100)
WWF-1	16a	20c	16a	13a	5b	70a
WWF-2	14b	20c	14b	11bc	4c	63b
WWF-3	12c	20c	12c	8d	3d	55c
WWF-4	16a	20c	14b	10c	6a	66b
WWF-5	6d	20c	10d	7d	2e	45d
RF	18	32	16	12	9	87

The scores were reported as mean values from five panelists. Within each column, values followed by different letters are significantly different (*p* < 0.05). WWF: whole-wheat flour, RF: refined flour. WWF-1: total reconstitution of brans grinded by hammer mill; WWF-2: total reconstitution of brans grinded by jet mill at 45 Hz. WWF-3: total reconstitution of brans grinded by jet mill at 65 Hz; WWF-4: entire grains grinded by jet mill at 45 Hz; WWF-5: entire grains grinded by jet mill at 65 Hz.

## Data Availability

The datasets generated for this study are available on request to the corresponding author.

## Supplementary Materials

The following are available online at https://www.mdpi.com/article/10.3390/foods10112857/s1, Table S1. Different milling methods used for preparing WWF, Figure S1. Particle size distributions of WWFs, Figure S2. Correlation analyses of sensory quality and chemical composition, Figure S3. Storage quality of CSB.
