# Supplementary material for: Effects of Different Pilot-Scale Milling Methods on Bioactive Components and End-Use Properties of Whole Wheat Flour
Source: Foods. 2021 Nov 18;10(11):2857. doi: 10.3390/foods10112857 (PMC8623663; doi:10.3390/foods10112857)
Supplement: Supplementary file 1 [file foods-10-02857-s001.zip › foods-1433530-supplementary.pdf]

Effects of different pilot-scale milling methods on bioactive components and end-use properties of whole wheat flour

Supplementary documents

Table S1. Different milling methods used for preparing WWF.

Sample	Flour type	Mill type
WWF-1	Total bran reconstitution	HM
WWF-2		JM-45 Hz
WWF-3		JM-65 Hz
WWF-4	Entire grain grinding	JM-45 Hz
WWF-5		JM-65 Hz

WWF: whole-wheat flour; HM: bran ground by hammer mill; JM-45 Hz: bran or grains pulverized by jet mill with the rotor speed of 45 Hz; JM-65 Hz: bran or grains pulverized by jet mill with the rotor speed of 65 Hz. WWF-1: Total reconstitution of brans grinded by hammer mill; WWF-2: Total reconstitution of brans grinded by jet mill at 45Hz. WWF-3: Total reconstitution of brans grinded by jet mill at 65Hz; WWF-4: Entire grains grinded by jet mill at 45Hz; WWF-5: Entire grains grinded by jet mill at 65Hz.

Figure S1. Particle size distributions of WWFs.

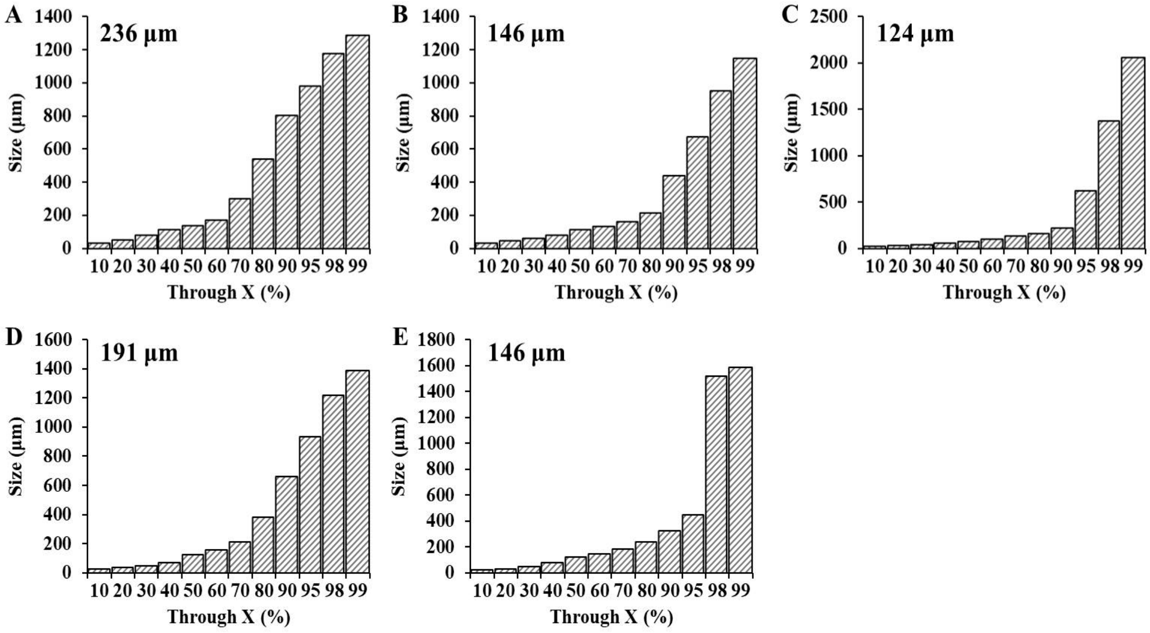

Figure A, B, C, D, E, particle size distributions for WWF-1, WWF-2, WWF-3, WWF-4, and WWF-5, respectively. The average particle size is noted in each figure.

Figure S2. Correlation analyses of sensory quality and chemical composition.

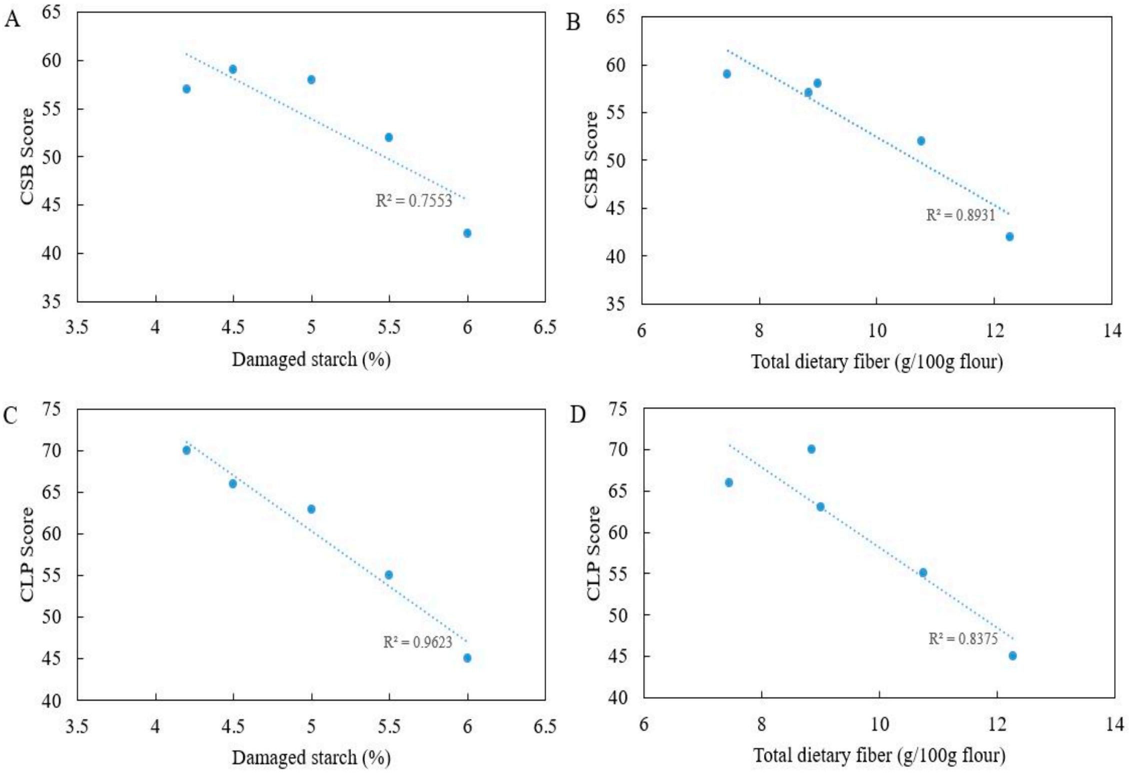

Correlations between (A) CSB score and damaged starch ($R^2 = 0.7553$), (B) CSB score and total dietary fiber ($R^2 = 0.8931$), (C) CLP score and damaged starch ($R^2 = 0.9623$), and (D) CLP score and total dietary fiber ($R^2 = 0.8375$).

CSB, Chinese steamed bread; CLP, Chinese leavened pancake

Figure S3. Storage quality of CSB

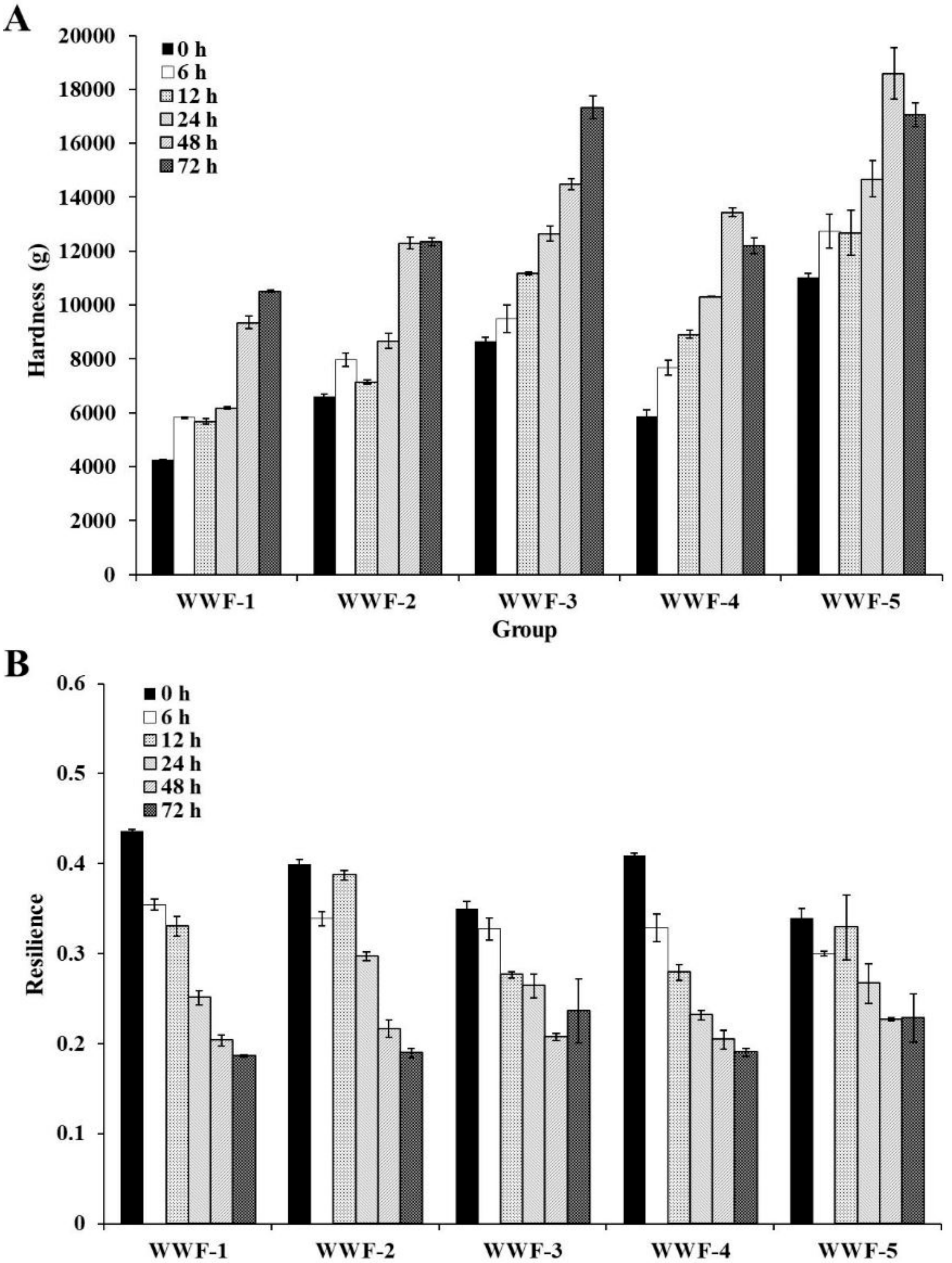

CSB, Chinese steamed bread. A, changes in hardness from 0 to 72 hours; B: changes of resilience from 0 to 72 hours.
